# A Culturally Responsive Curricular Revision to Improve Engagement and Learning in an Undergraduate Microbiology Lab Course

**DOI:** 10.3389/fmicb.2020.577852

**Published:** 2021-01-13

**Authors:** Karla S. Fuller, Camila Torres Rivera

**Affiliations:** ^1^Science Department, Stella and Charles Guttman Community College, City University of New York, New York, NY, United States; ^2^Mathematics Department, Stella and Charles Guttman Community College, City University of New York, New York, NY, United States

**Keywords:** curriculum, undergraduate, research, culturally responsive, pedagogy

## Abstract

We seek to increase student engagement and success to subsequently lead to increased retention and degree attainment for students at our Hispanic-serving institution. We hypothesized that using a culturally responsive approach in an undergraduate microbiology lab would increase engagement and learning gains. Using a culturally responsive approach allowed students to start their learning from their own place of understanding—centering students’ lived experiences. Students interviewed family members to learn about “home remedies,” and then devised experiments to test whether those home remedies affected growth of bacteria commonly implicated in gastrointestinal distress (*Staphylococcus aureus*, *Bacillus cereus*, and *Escherichia coli*) or sore throat (*Neisseria gonorrhoeae*, *Streptococcus pyogenes*, and *Mycoplasma pneumoniae*). As a final assessment, students generated project posters which they presented at a class symposium. Implementation of a culturally responsive research experience focused on the gut microbiome resulted in increased learning gains as evidenced by movement up Bloom’s Revised Taxonomy Scale. Student feedback indicated increased engagement, increased confidence in communicating science and a deeper understanding and appreciation for microbiology. Taken together, the results indicate that students appreciate a more culturally responsive and student-centered approach to learning in microbiology and encourages expansion of this approach to other modules in the course. This paper includes responsive data to support this claim, as well as a sample course calendar and supplementary learning material to support the human microbiome approach to microbiology.

## Introduction

Bacterial metabolism is a core concept for undergraduate microbiology education (Merkel and Reynolds, 2014). The complexity of this multi-dimensional topic can make it difficult for students to grasp core concepts in a meaningful way as they struggle to find the significance of these concepts relative to their lived experiences. On the other hand, antibiotic sensitivity has immediate “real-life” applications and students easily grasp the concept. Yet, the common ways to teach antibiotic sensitivity—through antibiotic disc or similar cookbook-style zone of inhibition lab experiments—can seem simplistic.

In our course assessments, students consistently demonstrate low engagement with the bacterial metabolism module and struggle to relate the concepts of bacterial metabolism to larger themes, such as the human health and physiology. Generally, students rate our antibiotic sensitivity module, low on the engagement scale, even as they meet the learning goals associated with the module. We sought to improve engagement and learning in these important themes in our undergraduate microbiology course by combining them under the overall topic of the human microbiome.

The human microbiome is a key influencer of our mental and physical health (Shreiner et al., 2015; Malan-Muller et al., 2017; Fitzgibbon and Mills, 2020). Recent studies test long-held claims that certain dietary plants contribute to health (Katoch et al., 2013; Praditya et al., 2019). As scientists learn more about the interactions between the food people consume and the microbiome, the role of these interactions in influencing a wide range of diseases, including obesity, allergies, metabolic diseases and cancer, becomes more evident (Gensollen et al., 2016). In the future, microbiome evaluation may become an important part of disease diagnosis and treatment. Therefore, it is important that emerging scientists begin to grasp these concepts now to understand the role of microbiome in maintaining health as medical science moves toward a more personalized approach to treatments.

As such, we sought to revise our microbiology curriculum to include microbiome-specific learning experiences. We merged two course modules (bacterial metabolism and antibiotic sensitivity) to create one curricular undergraduate research experience (CURE) in the accompanying lab course. We selected these modules because bacterial metabolism typically has low learning goals success rate and antibiotic sensitivity typically has low engagement rankings.

Several studies have described increased problem-solving abilities and critical thinking, when CUREs are implemented in the undergraduate biology classroom (Cooper et al., 2019; Hurst-Kennedy et al., 2020). The 2012 *Engage to Excel* report from the President’s Council of Advisors on Science and Technology encouraged discovery-based research experiences as part of STEM curriculum to increase student engagement and ownership of projects (Olson and Riordan, 2012). To ensure the CURE met the course learning outcomes, we adapted a backward design framework to build the CURE into the curriculum (Reynolds and Kearns, 2017). Our intervention seeks to increase learning gains and engagement through relevancy to improve the student learning experience.

To make the content more responsive, we incorporated culturally responsive teaching (CRT) into the CURE design process (Aronson and Laughter, 2016; Cooper et al., 2017; Gay, 2018). In particular, the teaching goals are discreetly outlined to use cultural knowledge as context for the research project (Table 1). The initial background knowledge for the CURE is gathered using ethnographic interviews of family members. Using techniques of ethnographic research ground the researcher’s approach in cultural knowledge and awareness and place social knowledge in parallel prominence with scientific knowledge (Atkinson and Pugsley, 2005). This approach, using documented family knowledge for direct engagement in a microbiome research question, has been described previously with success in increased engagement by Pérez-Losada et al. (2020). In their study, students compared skin microbiome composition using a “grandma hypothesis”—areas of the body that grandmothers are always telling their grandchildren to clean carefully, such as behind the ears and between the toes.

**TABLE 1:** Example of mapping one outcome according to CURE Backward design process with embedded culturally responsive teaching (CRT) strategies.

Backward design step	Scientific discovery	CRT practice
Identify desired outcomes	Students determine if selected foods have antimicrobial properties	• Affirm that cultural traditions have ways of knowing. • Cross-cultural exchange from student-student
Determine acceptable evidence	Students measure zone of inhibition from food samples, using antibiotics as controls	• Student as knowledge holder—professor as facilitator • Scaffolded assignment with clear expectations at each step
Planned learning experiences and instruction	(1) Interview family knowledge holders (2) Culture bacteria (3) Measure CFU from serial dilution (4) Design and implement zone of inhibition study to test food item(s)	• Learning within the context of culture • Students research aspects of the topic in their community • Students share their work with their family/community using culturally appropriate language

**Using the backward design process ensured that the introductory labs provided sufficient training and knowledge for student independence during the research project. THE CRT practice principles were visible in each outcome of the research project.**

The CRT framework increases access to the skills, knowledge, and competencies necessary for success (Aronson and Laughter, 2016). This is a high priority in our upper-division biology courses, as success in these courses correlates to post-graduation transfer and persistence in science programs at 4-year colleges (Woods, n.d.). Culturally responsive teaching embraces cultural knowledge, personal frames of reference, and lived experiences to make learning more responsive to and effective for students (Snively and Corsiglia, 2001; Hernandez et al., 2013).

Research studies have also described a retention and belongingness benefit to undergraduate CUREs for students from underrepresented groups (Bangera and Brownell, 2014; Elgin et al., 2016). As the majority of our students identify as Black and/or Latino, both CRT and CURE promised benefits uniquely suited to the success of our student population.

In a previous study, Skendzic and Keler (2019) describe a fruit fly microbiome CURE in which students supplemented fruit fly media with various antibiotics, probiotics, and common food additives (i.e., monosodium glutamate and food dye), then assessed the effect of these supplements on the growth of *Lactobacillus*, the most prevalent bacteria in the fruit fly gut. We have extended this type of inquiry-based experiment by adding a personal component.

In the CURE we describe in this paper, students used principles of ethnographic research to engage in family interviews to determine which variables (home remedies) to include in their research. This data was paired with what they have learned in the antibiotic resistance module to set up a comparative study. Thus, in addition to studying the effects of purported antibacterial substances on common bacterial populations, students could make direct connections between the microbiome and overall health.

The inclusion of ethnographic research as the foundation of a series of student-derived experiments allows students to demonstrate their understanding of bacterial metabolism and antibiotic sensitivity while demonstrating mastery of bacterial culture techniques in a project with a personal connection. We hypothesized that a CRT-CURE intervention would increase engagement with these topics by making them more responsive to students and this increased relevancy would promote learning gains. Furthermore, we speculated increased engagement in the course might alleviate some of the ostracizing learning experiences underrepresented students often face in science classes by centering the context of the material around their own communities and cultural practices. This study builds on the success of other well-known CURES, such as the Bean Beetle Microbiome CURE, by adding a personal component to an authentic research experience (Zelaya et al., 2020). Having students combine cultural knowledge with microbiology theory to create a novel research question adds value to the educational process and traditional practices and can begin to dismantle traditional modes of formal education, engaging a diverse student body (Baines and Zarger, 2017).

## Materials and Methods

### The Assignment

Students learned typical microbiology lab techniques during the first 6-weeks of the lab (hereafter referred to as “introductory labs”). At the end of each introductory lab module, students completed a two-question engagement survey (Table 2). For homework, students completed Thought Questions related to the lab on an adaptive learning platform (Table 3). The questions were scaled according to Bloom’s Revised Taxonomy Scale, with the first question from the remembering domain and the subsequent questions moving up the scale through the understanding, applying, and analyzing domains (Krathwohl, 2010).

**TABLE 2:** After each introductory and CURE lab, students completed the engagement survey below anonymously.

	1—Strongly disagree	2—Disagree	3—Neither agree nor disagree	4—Agree	5—Strongly agree
This lab was interesting					
I used my own creativity during this lab.					

**TABLE 3:** Post-lab Thought Questions were deployed using adaptive learning software after each introductory and CURE lab.

Lab topic	Domain: remembering	Domain: understanding	Domain: applying	Domain: analyzing
Lab 1—Bacterial Staining; Identify bacteria by morphology	1. Label the parts of the bacterial cell on the drawing below. 2. Create a chart to organize the staining techniques and the types of bacteria they stain, including predicted color results*.	3. Classify the bacteria below according to morphology and. 4. Classify the bacteria according to stain.	5. A spore has a mutation that prevents the production of keratin in the membrane, describe how this would affect the results of the spore stain test.	
Lab 2—Aseptic Technique—streak plates, inoculate liquid cultures	1. Predict the results from your Kirby-Bauer experiment.	2. Make a comparison chart of the types of selective media used in today’s experiment. 3. Based on your experimental data, which antibiotic is most effective for your given bacterial sample.	4. Which media would you use to culture [a given bacteria]?	5. Using the class data, which antibiotic is most effective for many types of bacteria? Which has the least effectivity?
Lab 3—Serial dilutions; antibiotic sensitivity and the Kirby-Bauer test		1. Explain why serial dilutions are important for bacterial cultures.	2. Given a sample with known concentration X, design a serial dilution to produce final concentration Y.	3. Use the given set of results to match the bacterial plates with the serial dilution tube.
Lab 4—Identify bacteria by biochemistry	1. Students fill in a chart including names of test, brief description of test, examples of bacteria that would test positive*.	2. Given a data set, students can identify the type of bacteria used in the test.	3. Plan and conduct an experiment using the test you have been assigned.	4. Using class data, identify the unknown bacteria.
Lab 5—Bacterial metabolism/cellular respiration	1. Compare and contrast fermentation and cellular respiration	2. When would a metabolically versatile microbe perform fermentation rather than cellular respiration?	3. Can protein catabolism help identify microbes?	

**Each question series begins with a question from the Remembering domain. If students answered a question incorrectly, they were automatically exited from the question series. ^∗^Question not included in CURE lab and omitted from statistical analysis.**

Only if students answered a question correctly would they see the next question in that series. If students answered a question incorrectly, the session would end automatically. Once students have learned the necessary techniques and background knowledge through the introductory labs, they embark on a carefully scaffolded research project that has four main components: (1) interview, (2) library database research, (3) lab research, and (4) culminating research report. Standard biosecurity and institutional safety procedures were adhered to during this lab course.

The CURE began in Week 7 with a simple question: If you had a stomachache or a sore throat, who in your family would you ask for a remedy? Students interview a family member about a home remedy for an upset stomach and/or a sore throat, then use the ethnographic interview to generate a set of observations. Interviewing family members about health practices provided the real-world context that is important for making long-lasting curricular connections. Students recorded their interviews and organized them into a set of observations they could use as the basis for research to find preliminary scientific data to support the claims of the knowledge-holder. For instance, a grandmother describes a baking soda-honey solution she uses to soothe a sore throat, which prompts a student to search library databases to find sources related to the antimicrobial properties of sodium bicarbonate and honey.

After students completed their interviews, we used class discussions to generate common research questions. The class agreed on the following research questions: (1) Does the home remedy kill bacteria, or does it stop the symptoms? and, (2) How does the home remedy compare to antibiotic treatment? Similarly, we agreed on common class hypotheses: H_1_—The food additive inhibits bacterial growth; H_0_—The food additive does not inhibit growth. Interestingly, in class discussion, students agreed that even if the food additive does not inhibit growth, it may be effective in treating the symptoms. Bacteria commonly implicated in gastrointestinal distress (*Staphylococcus aureus, Bacillus cereus*, and *Escherichia coli*) or sore throat (*Neisseria gonorrhoeae, Streptococcus pyogenes*, and *Mycoplasma pneumoniae*) were used in the study.

As a final step in the pre-planning process, students generated individual hypotheses for their experiments, which were validated by peer-review (Ex. I hypothesize that chamomile tea reduces bacterial growth). Then students designed an experiment to test their hypotheses. Before starting experimentation, students met individually with the instructor for experimental design approval. Afterward, students were free to conduct their experiments at their own pace, within the allotted 4-week time period. To manage a course with 23 concurrent independent experiments, we created a lab schedule for certain tests. For instance, students would plan to have their experiment complete and a sample set ready for gram staining during Week 9 when the supplies and materials for gram staining were available in the lab (Table 4). After the weekly experimentation was done, students had a second attempt at the previous Thought Questions on the same adaptative learning platform. To conclude the research experience, students created research posters to present their projects to the campus community (Supplementary Figure 1). Thus, in our experimental design, the introductory labs served as the control group and the CURE intervention served as the experimental group.

**TABLE 4:** CRT-CURE lab course calendar with associated tasks.

Week	Lab topic	Research project task
1	Identify bacteria by morphology Bacterial staining lab (gram and acid-fast stain)	Project Introduction and overview (no tasks)
2	Aseptic technique Kirby-Bauer test	Ethnographic interviews
3	Serial dilutions and growth curves	Class data gathering
4	Biochemical testing to identify bacteria	Information Literacy (library research)
5	Cellular respiration and fermentation	Research Question brainstorm
6	Research project	Hypothesis forming
7		Research plan review and approval
8		Serial dilutions and growth curves Kirby-Bauer test
9		Gram-staining Biochemical testing
10		Cellular respiration and fermentation test
11		Research report writing time
12		Class symposium

**Weeks 1–5 are introductory skills-building labs. In weeks 8–10, students use the skills they learned in the introductory modules for their own experimentation.**

It is also important to mention as part of the assignment, students gave consent for the instructor to submit on their behalf their research project abstracts for conference presentations. Two projects were accepted for the student e-poster competition at the 2019 American Association for the Advancement of Science (AAAS) Annual Meeting.

### Survey Methods

Student engagement surveys were conducted anonymously and collected on a single tablet via Google Forms at two points in the semester—immediately after each introductory lab and during the CURE. The introductory labs were traditional guided inquiry labs that focused on skill-building (how to use the tools, do the technique appropriately, and analyze the type of data generated by the technique accurately). Students responded to two prompts: “This lab was interesting,” and “I used my own creativity during this lab” using a Likert scale where 1 = strongly disagree, 2 = disagree, 3 = neither agree nor disagree, 4 = agree and 5 = strongly agree. *Strongly agree* and *agree* categories were aggregated to indicate a positive engagement response. *Strongly disagree* and *disagree* categories were aggregated to indicate a negative engagement response.

For the Thought Questions, pre- and post-intervention grades were paired, then anonymized by a research assistant and analyzed after the course was over. De-identified open-ended comments were collected through student evaluations at the end of the course.

### Data Analysis Methods

De-identified, raw data was provided by Dr. Fuller in a Microsoft Excel spreadsheet. The data was comprised of two, parallel satisfaction items that were measured using a five-point Likert scale. The five response categories for the Likert scale were; 1- strongly disagree, 2- disagree, 3- neither agree nor disagree, 4, agree, 5 strongly agree. The pre-condition (SatisReg) satisfaction responses (*n* = 234) were collected over the first five lab sessions. Post-condition (SatisCURE) responses were collected for the five lab sessions (*n* = 240) following the intervention.

Using SPSS, the Likert scaled data was entered and coded according to the point values of each response category. Four new variables (i.e., SatisRegAlla, SatisRegAllb, SatisCUREAlla, SatisCUREAllb) were created by calculating the sum of all the Likert scores for cases with a full set of responses (*n* = 21). Numerical and graphical assumption tests were performed on the newly transformed variables, and all assumptions for normality were met. The means of the variables were analyzed using right, one-tailed, paired *t*-tests (α = 0.05) to determine if there was a statistically significant increase between the means of the pre- and post-groups for each of the two questions.

De-identified raw data from the Thought Questions was collected and coded as *correct* vs. *incorrect* and *did not attempt* vs. *attempt*. In addition to the raw scores, Thought Question scores were categorized according to the first four levels of Bloom’s Revised Taxonomy scale (i.e., Remembering, understanding, applying, and analyzing). Using SPSS, the data was entered and coded. Assumptions for normality were met by the Central Limit Theorem. Two-proportion *t*-tests (α = 0.05) were performed comparing introductory lab data and CURE data for the same Thought Questions. We then analyzed the number of correct responses and question attempts along the Bloom’s Revised scale after the CURE intervention.

## Results

We used student interest to measure engagement. Student interest in lab work increased during the CURE, compared to their interest in the introductory labs. Students also reported the CURE lab structure allowed them to be more creative in their experiments (Table 5).

**TABLE 5:** Assessment of student engagement.

	Introductory Labs	CURE labs
This lab was interesting (strongly agree/agree)	16.9	21.9^a^
I used my own creativity during this lab (strongly agree/agree)	11	19.8^b^

**Student engagement in the introductory and CURE labs was measured by anonymous survey responses to the prompts to gauge student interest and creativity. Mean scores for strongly agree/agree were aggregated and compared between introductory and CURE post-lab surveys. ^*a*^SD = 1.7, p < 0.0001. ^*b*^SD = 1.4, p < 0.0001.**

**FIGURE 1:**
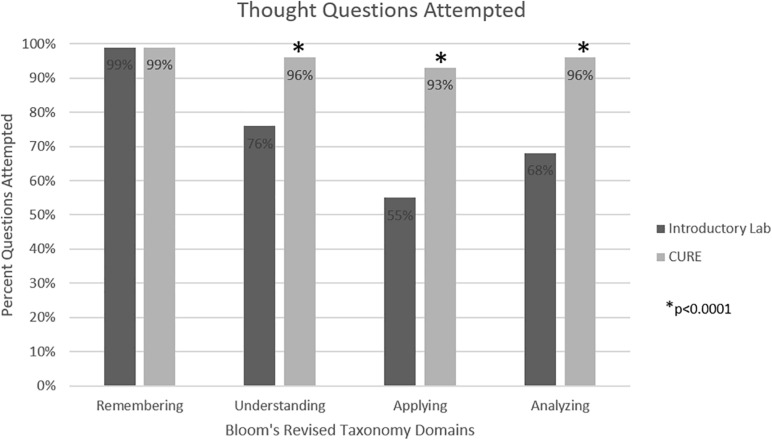
Attempted responses. Comparison of the percent of students who attempted Thought Question at each domain of the Bloom’s Revised Taxonomy scale (remembering) after the introductory and CURE labs. *p < 0.0001.

**FIGURE 2:**
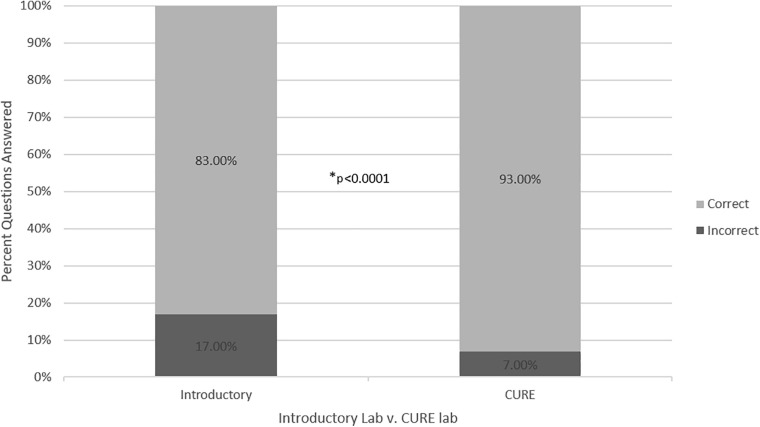
Correct answers. Comparison of the proportion of correct answers per each Thought Question attempt after the introductory and CURE labs. *p < 0.0001.

To measure learning gains, at the end of each introductory module, students were given a series of questions that moved progressively up the first four levels of Bloom’s scale: (1) remembering, (2) understanding, (3) applying, and (4) analyzing. Students were given the same set of questions after each related experiment during the CURE. More students attempted questions in the higher domains (understanding, applying, and analyzing), suggesting that students were more confident to try the more difficult questions after doing the experiments in the context of the CURE (Figure 1). Overall, students answered more Thought Questions correctly during the CURE than during the introductory labs (Figure 2).

During the end-of-semester student evaluations, we asked students to tell us if the overall course experience changed their views of themselves as scientists. Wherein, we anticipated students to mention a growth in skills and ability, many comments addressed a deeper appreciation for the nature of science and what it means to be a researcher (Table 6).

**TABLE 6:** Student feedback from open-ended question: “Has the research project changed your perceptions of doing science?”

Before I used to get mad when my experiments didn’t work. But because we had more time to work through our problems during the research project, I feel like I learned from my mistakes.
I feel like my family respects my career goals more. When I talked to them about my project, they were interested and impressed that I was using microbiology to test my grandmother’s remedies.
I feel like I have a better understanding of how research fits into like general medical care. There is a lot of work that would go into creating one medical treatment.
[The instructor] is always calling us scientists and specialists, but this time I really felt like I was a scientist!
Working on my own research helped me grow as a scientist.
Even though I was nervous to do the project on my own, I felt more confident because we could use each other as collaborators. For once we were talking about our research when we met in the commons instead of just chatting about nothing.
I never thought I could design a project, do the research, then create a good poster. This is also the first time that I was asking questions during presentations because I really wanted to know the answers not just to get the participation grade.
I had a really hard time getting my experiments to work but [the instructor] kept encouraging me and in the end I figured it out. I don’t think I’ll ever do scientific research as a career, but I do feel more confident for the next classes because I know if I take my time and follow my mistakes I can figure it out.
Even though we worked on individual projects, I really felt like we were a team. We had to peer-review each other’s papers and [the instructor] made us ask each other for help before we asked her so everybody was really helping each other.
For once I see that the things I’m learning in my classes will actually help me when I get a career. Like, I can really do these experiments if I got this kind of job. And I have a whole project in my ePortfolio that proves it.
I never really had a connection with the research projects and experiments in my classes. I want to be a doctor so I can help my community but this project has me thinking about ways that different types of scientists can help communities. I never thought about it like that before.

**“This lab was interesting.”**

The mean of the post-condition group ($\bar{x}$ = 21.9; *SD* = 1.7) was greater than the mean of the pre-condition group ($\bar{x}$ = 16.9; *SD* = 2.3). There was a statistically significant increase in students’ perceived interest in the labs from the pre-condition group to the post-condition group [*t*(20) = 7.3, *p <* 0.0001, right one-tailed, *t*-test]. Results show that there is enough evidence to reject the null hypothesis of no difference.

**“I used my own creativity during this lab.”**

The mean of the post-condition group ($\bar{x}$ = 19.8; *SD* = 1.4) was greater than the mean of the pre-condition group ($\bar{x}$ = 11.0; *SD* = 1.9). There was a statistically significant increase in students’ perceived use of their own creativity in the labs from the pre-condition group to the post-condition group [*t*(20) = 16.54, *p <* 0.0001, right one-tailed *t*-test]. Results show that there is enough evidence to reject the null hypothesis of no difference.

## Discussion

This study had two pedagogical goals—to increase student engagement and to increase student learning outcomes. We addressed these two issues by focusing on using an inquiry-based microbiome study driven by student-generated questions. An ideal laboratory project allows students to: (1) apply theory to practice, (2) use critical thinking to apply the scientific process, and (3) increase interest and motivation (Nicolaidou et al., 2019).

Success with culturally responsive pedagogies has been well-documented in K-12 education. However, there is a lack of published studies outlining the success and implementation guidance on CRT at the undergraduate level, particularly in the sciences.

On the other hand, there is much data to support CUREs as a beneficial intervention in undergraduate biology with reports documenting improvements in learning, belongingness, and retention (Rainey et al., 2018; Nerio et al., 2019). Here we describe a culturally responsive CURE that combines the engagement benefits of CRT and the learning benefits of CURE as a successful model for teaching and learning in an undergraduate microbiology course.

In addition to the learning gains, or perhaps in support of them, this course re-design gave more opportunities for formative assessment because of the close scaffolding of the research project, one-on-one meetings for research design approval, and pre- and post-proposal student/student peer-review. In the end, students were able to relate the concept of the microbiome to their personal experiences by learning about human gut bacteria commonly implicated in illnesses and how bacterial populations are affected by the foods we eat.

Asking students to design experiments based on family stories is quite a shift in the way we normally teach microbiology. In doing so, we had to step outside of the normal formula of “teach, show, do” to evolve the learning experience by incorporating a deeper exploration of topics in a skills-based environment across the entire semester. Introducing students to theory in lecture, accompanied by demonstrations and training in lab are common for undergraduate science courses and can certainly get students to meet the course learning outcomes. However, stretching the student experience by deepening learning through student-generated research has additional benefits. The use of ethnographic interviews to gather initial observations allows a constructivist approach to teaching. This shifts the instructor-student relationship from one of knowledge holder (instructor) and knowledge receiver (student) to one that places students as active knowledge constructors and allows the instructor to act as facilitator (Lee and Hannafin, 2016). Thus, the aim of this project is to re-center the learning experience around the student by breaking away from the cookbook model of laboratory science and facilitating an outward learning process that has the students’ lived experience at the core in an effort to strengthen engagement.

Due to the success of this intervention, we are currently re-designing two additional courses (one statistics course and one introduction to biology course) to include a CRT-CURE intervention. We intend to continue strategic course re-designs so that each student in the science program will have at least two CRT-CURE courses as they matriculate through the Associate’s Degree program.

## Data Availability Statement

All datasets generated for this study are included in the article/Supplementary Material, further inquiries can be directed to the corresponding author.

## Ethics Statement

This study involving human participants was reviewed and approved by the City University of New York Institutional Review Board (IRB# 2020-0324). Student participants gave written informed consent prior to this study.

## Author Contributions

KF conceived of and implemented the study and wrote the manuscript. CT carried out the data analysis for the manuscript. Both authors contributed to the article and approved the submitted version.

## Conflict of Interest

The authors declare that the research was conducted in the absence of any commercial or financial relationships that could be construed as a potential conflict of interest. The reviewer MT declared a shared affiliation with several of the authors, KF and CT, to the handling editor at the time of the review.

## Biosafety

This project was performed in a Biosafety Level 1 laboratory following all of the required safety measures for handling and disposal of biological agents using non-infectious strains of the aforementioned biology.

## Supplementary Material

Supplementary Figure 1 Sample student poster.
