# Supplementary figures and images for: A Culturally Responsive Curricular Revision to Improve Engagement and Learning in an Undergraduate Microbiology Lab Course
Source: Front Microbiol. 2021 Jan 13;11:577852. doi: 10.3389/fmicb.2020.577852 (PMC7838382; doi:10.3389/fmicb.2020.577852)

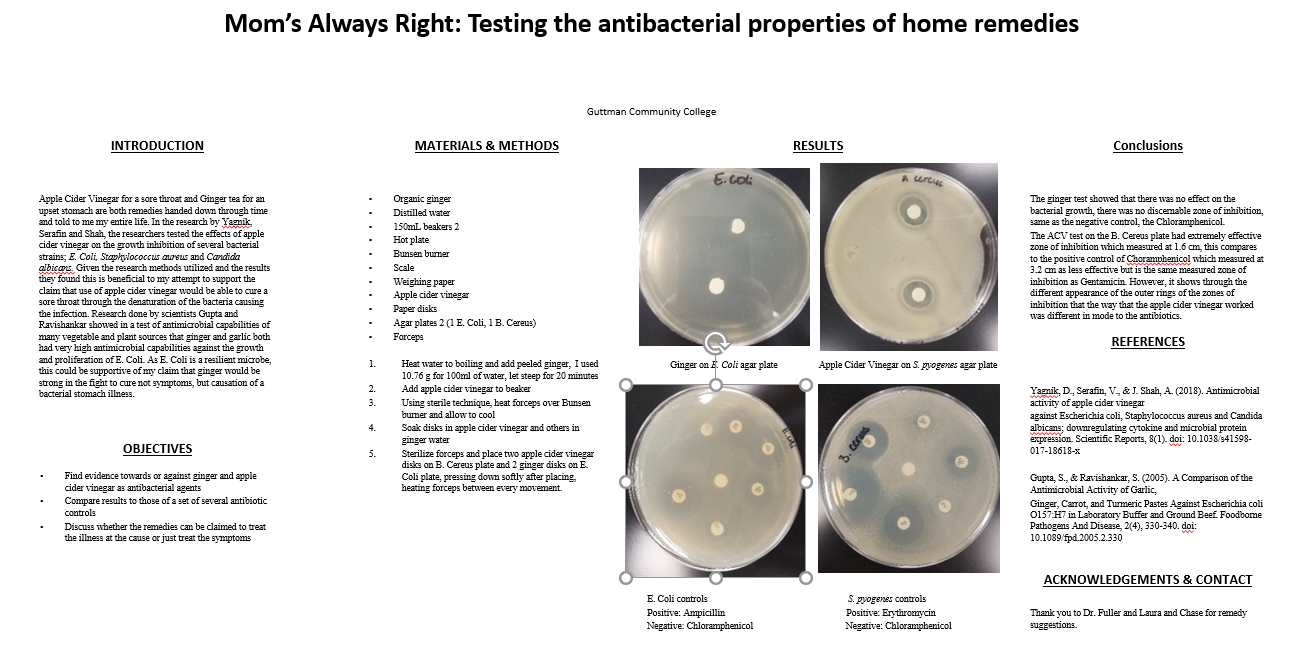

Supplement: Supplementary Figure 1 — Sample student poster. [file Image_1.PNG]
